# Evaluation of Extraction Protocols for Simultaneous Polar and Non-Polar Yeast Metabolite Analysis Using Multivariate Projection Methods

**DOI:** 10.3390/metabo3030592

**Published:** 2013-07-23

**Authors:** Nicolas P. Tambellini, Vanina Zaremberg, Raymond J. Turner, Aalim M. Weljie

**Affiliations:** 1 Department of Biological Sciences, University of Calgary, 2500 University Drive NW, Calgary T2N 1N4, AB, Canada; E-Mails: nprtambe@ucalgary.ca (N.P.T.); vzarembe@ucalgary.ca (V.Z.); turnerr@ucalgary.ca (R.J.T.); 2 Metabolomics Research Centre, University of Calgary, 2500 University Drive NW, Calgary T2N 1N4, AB, Canada; 3 Department of Pharmacology, University of Pennsylvania Perelman School of Medicine, 34th & Civic Center Boulevard, Philadelphia, PA 19104-5158, USA

**Keywords:** metabolite extraction, chloroform/methanol/water partitioning, GC-MS, fatty acid methyl esters, polar metabolites, PCA, OPLS, chemoinformatics

## Abstract

Metabolomic and lipidomic approaches aim to measure metabolites or lipids in the cell. Metabolite extraction is a key step in obtaining useful and reliable data for successful metabolite studies. Significant efforts have been made to identify the optimal extraction protocol for various platforms and biological systems, for both polar and non-polar metabolites. Here we report an approach utilizing chemoinformatics for systematic comparison of protocols to extract both from a single sample of the model yeast organism *Saccharomyces cerevisiae*. Three chloroform/methanol/water partitioning based extraction protocols found in literature were evaluated for their effectiveness at reproducibly extracting both polar and non-polar metabolites. Fatty acid methyl esters and methoxyamine/trimethylsilyl derivatized aqueous compounds were analyzed by gas chromatography mass spectrometry to evaluate non-polar or polar metabolite analysis. The comparative breadth and amount of recovered metabolites was evaluated using multivariate projection methods. This approach identified an optimal protocol consisting of 64 identified polar metabolites from 105 ion hits and 12 fatty acids recovered, and will potentially attenuate the error and variation associated with combining metabolite profiles from different samples for untargeted analysis with both polar and non-polar analytes. It also confirmed the value of using multivariate projection methods to compare established extraction protocols.

## 1. Introduction

The field of metabolomics is rapidly seeing more widespread use for the determination of system level metabolic changes caused by influences such as diet, environmental stress and disease [1,2,3,4,5]. However, to accurately determine the changes in a metabolite profile caused by these influences, care must be taken in order to optimize the different factors that affect data quality and reproducibility. Of paramount importance in this regard is the metabolite extraction step, as it affects both the number of different metabolites available for analysis as well as the reproducibility and reliability of the data obtained.

Studies looking at optimal polar metabolite extraction protocols for biological compounds including sugars, amino acids and water soluble metabolic precursors or intermediates have been carried out for single platforms including nuclear magnetic resonance (NMR) [6,7], gas chromatography mass spectrometry (GC-MS) [8,9,10,11], and liquid chromatography mass spectrometry (LC-MS) [12,13,14]. Additional studies have focused on a polar metabolite extraction for multi-platform use [15,16]. Optimal extraction protocols have been tested for biological samples including serum [13] and plasma [8], and for different model organisms such as *Escherichia coli* [9], *S. cerevisae* [17,18] and *Caenorhabditis elegans* [16]. Furthermore, some effort has also gone into determining the best extraction method for non-polar metabolites, such as fatty acids and other lipids [19,20,21], with these types of studies becoming more frequent as the subfield of metabolomics (also known as lipid profiling or lipidomics) has become more popular. For instance, detailed protocols on how to extract and analyse lipids from yeast [22], body fluids and tissues [23] are now available.

Though much work has gone into establishing protocols to look at either polar (water soluble) or non-polar (soluble in organic solvents) metabolites, little work has gone into finding a protocol that is effective at simultaneously extracting both polar and non-polar metabolites. Analysis of both polar and non-polar metabolites from the same samples could be extremely beneficial in future metabolomic studies, as it will avoid much of the variation that can occur when trying to combine both types of metabolite information from separate samples. Additionally, much of the previous work with optimizing extraction of metabolites has centered on comparing different types of quenching and extraction solvents, while focusing mainly on optimal metabolite recovery as opposed to reproducibility.

Here we investigated three different chloroform/methanol/water based metabolite extraction protocols found in the literature on *S. cerevisiae* for the ability to reproducibly extract high levels of polar and non-polar fatty acid metabolites. Chloroform/methanol/water based protocols were explored as they are generally the standard for classical lipid/fatty acid extractions [24,25] and have had success extracting polar metabolites in yeast [18], among other sample types. Yeast was used as it is accepted as a suitable fungal representative of the microbial community, and as a model system for eukaryotic organisms. Additionally, it is unique in containing only mono-unsaturated and even numbered fatty acid chain lengths, thus simplifying analysis. We were able to successfully identify a protocol using chemoinformatics and multivariate projection methods that was able to reproducibly extract comparatively high levels of both polar metabolites and non-polar fatty acids.

## 2. Results and Discussion

Cheminformatics is a field that is growing rapidly and will be of great use for metabolomic studies, especially as compound databases continue to expand. This will allow for untargeted analysis of many different sample types to be carried out. As more untargeted metabolomic studies are done, it becomes necessary to use multivariate projection methods—such as those discussed below—to help interpret the complex data collected. Using this approach we can apply metabolomics in many different areas, including biomarker studies, drug monitoring, identifying effects of diet and responses to external stresses or stimuli.

As metabolomics becomes a more prevalent tool across multiple scientific disciplines, it has become clear that the extraction protocol used has a large influence on the quality and reliability of the data obtained. As such, numerous studies have tried to improve upon previously accepted metabolite extraction methods, and identify an optimal extraction protocol for various platforms [6,7,8,9,10,11,12,13,14] and sample types [8,9,13,16,17,18]—though few, if any, have attempted to systematically identify an extraction protocol that is able to obtain both polar and fatty acid/lipid metabolites simultaneously. Additionally, most of these studies have focused on optimizing the percent metabolite recovery through the use of internal standards, with less focus placed on the reproducibility of the extraction protocol being utilized. As there are now a large number of validated optimal extraction protocols suggested for various platforms and biological samples, it must be recognized that obtaining reproducible extraction from sample to sample is also of significant concern and should be explored further. We also believe that it is important to recognize that biological stresses and disease affect both polar and non-polar metabolites in organisms or biological samples, and extraction of both types of metabolites from the same sample will overcome much of the variation and drift problems associated with combining data from different samples. Typical problems leading to these undesirable effects can include small changes in atmospheric pressure, temperature and growth media composition that occur between cultures. Additionally, the use of different extraction techniques and solvents can lead to metabolite loss and/or variation or drift between samples reducing reliability and reproducibility.

### 2.1. Unsupervised Analysis Clearly Differentiates Extraction Method

**Figure 1:**
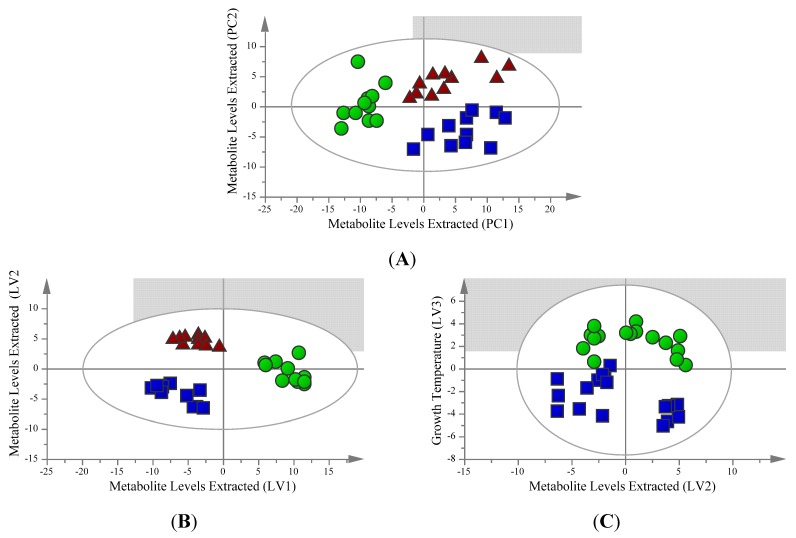
Models for polar metabolites extracted from *S. cerevisiae* by one of three chloroform/methanol/water based extraction protocols. An overview of the data confirms that there are no outlying samples within a 95% confidence interval. (**A**) PCA scores plot model with *R*^2^ = 0.557 and *Q*^2^ = 0.438 values. Green circles represent samples extracted using method 1, blue squares represent samples extracted using method 2 and red triangles represent samples extracted using method 3. (**B**) Orthogonal partial least squares discriminant analysis (OPLS-DA) scores plot model of predictive latent variables (LV) 1 and 2 showing separation based on extraction method with *R*^2^(X) = 0.704, *R*^2^(Y) = 0.933, *Q*^2^ = 0.881 and cross validated analysis of variance (CV-ANOVA) *p* = 4.20 × 10^−7^ values. Green circles represent samples extracted using method 1, blue squares represent samples extracted using method 2 and red triangles represent samples extracted using method 3. (**C**) OPLS-DA scores plot model of predictive latent variables (LV) 2 and 3 showing separation based on growth temperature with *R*^2^(X) = 0.704, *R*^2^(Y) = 0.933, *Q*^2^ = 0.881 and CV-ANOVA *p* = 4.20 × 10^−7^ values. Green circles represent samples grown at 30 °C, blue squares represent samples grown at 37 °C.

**Figure 2:**
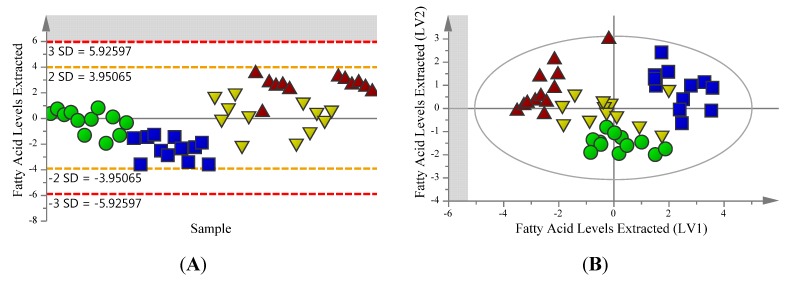
Models for fatty acid metabolites extracted from *S. cerevisiae* by one of three chloroform/methanol/water based extraction protocols. Fatty acid methyl esters (FAME’s) were identified through comparison to a 37 FAME standard. An overview of the data confirms that there are no outlying samples within a 95% confidence interval. Green circles represent samples extracted using method 1, blue squares represent samples extracted using method 2, red triangles represent apolar (17:1 CHCl_3_:MeOH) organic fraction samples extracted using method 3 and yellow upside down triangles represent polar (2:1 CHCl_3_:MeOH) organic fraction samples extracted using method 3. (**A**) PCA scores plot model with *R*^2^ = 0.325 and *Q*^2^ = 0.176 values. (**B**) OPLS-DA scores plot model showing separation based on extraction method with *R*^2^(X) = 0.616, *R*^2^(Y) = 0.517, *Q*^2^ = 0.411 and CV-ANOVA *p* = 1.48 × 10^−6^ values.

In order to assess the influence of the metabolite extraction process on both polar and non-polar metabolites, three separate procedures based on chloroform/methanol/water partitioning that have been used previously in the literature [26,27,28] were compared using multivariate modelling. The multivariate modelling was carried out with the polar metabolite extracts having a dimensionality of 33 samples with 322 total ion hits obtained, and lipid extracts having a dimensionality of 48 samples with 12 fatty acids monitored. Unsupervised principal component analysis (PCA) is a tool which provides a multivariate overview of the data based on the underlying variance between the metabolite profiles of the samples without specifying the different sample types. Analysis of this type is useful for screening of outliers and overviewing the metabolite patterns of the different sample types. Samples extracted for polar metabolites were screened for outliers with PCA (Figure 1A). The overall sample pattern shows clustering by extraction protocol and not by growth temperature, with *R*^2^ and *Q*^2^ values of 0.557 and 0.438 respectively for the model, which is within the acceptable range for a biological model. Samples extracted for fatty acids using fatty acid methyl ester (FAME) analysis were also examined for outliers using PCA (Figure 2A). The model consists of one component with *R*^2^ and *Q*^2^ values of 0.325 and 0.176 respectively, shows no outliers and also shows loose clustering of the samples based on the extraction protocol used.

### 2.2. Supervised Analysis Identifies 36 Metabolites and Four Fatty Acids Differentiating the Extraction Methods

In order to specifically identify which metabolites significantly contributed to the separation between sample groups, OPLS-DA was also performed on the dataset. OPLS-DA is a supervised analysis method to cluster multivariate data by maximizing the variance between different sample groups. OPLS-DA modelling of polar metabolite data produced an excellent three component model with *R*^2^(X) = 0.704, *R*^2^(Y) = 0.933, *Q*^2^ = 0.881 and CV-ANOVA *p* = 4.20 × 10^−7^, with all samples clustering into their respective extraction condition (Figure 1B). The first latent variable (LV) shows the separation of samples from Extraction 1 and the samples of Extractions 2 and 3, and the second latent variable shows separation of samples from Extractions 2 and 3. The third latent variable separates the samples based on their growth temperatures of 30 °C and 37 °C, with the samples of the same growth temperature clustering based on the protocol by which they were extracted (Figure 1C).

The OPLS-DA model for FAMEs shows the separation of Extraction 2 samples from Extraction 3 Polar and Extraction 3 Apolar samples with the first latent variable, while the second latent variable shows separation of Extraction 1 samples from Extraction 2 and Extraction 3 Apolar samples (Figure 2B). The model has a *R*^2^(X) = 0.616, *R*^2^(Y) = 0.517, *Q*^2^ = 0.411 and CV-ANOVA *p* = 1.48 × 10^−6^, and shows loose clustering of the samples based on the protocol with which they were extracted, with the exception of the Extraction 3 Polar samples which mix with each of the samples from the other extractions.

**Figure 3:**
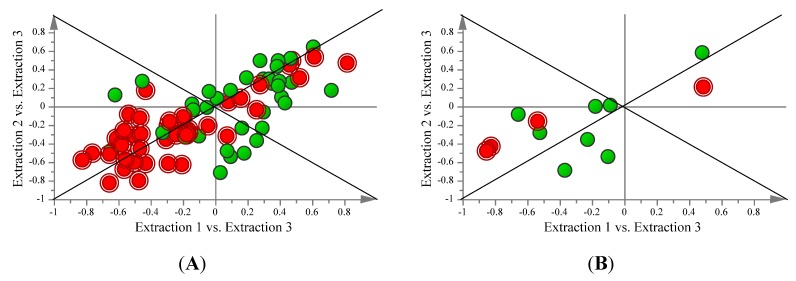
Shared and unique structure (SUS) plots of fatty acid and polar metabolites from *S. cerevisiae* by one of three chloroform/methanol/water based extraction protocols. Extraction 1 and Extraction 2 are plotted against Extraction 3 in order to observe the shared and unique metabolites obtained with Extractions 1 and 2. Red metabolites are those with a VIP greater than 1 as identified by OPLS-DA modelling. (**A**) SUS plot of polar metabolites. (**B**) SUS plots of FAME metabolites.

From OPLS-DA modelling, additional information can be obtained with regard to the metabolites contributing to the separation of sample groups and comparative metabolite levels using variable influence on projection (VIP) scores and coefficients. Comparing metabolites with a VIP score above 1 with the corresponding coefficient values for these metabolites from each extraction method gives a basis to compare the differences in metabolite levels extracted. Metabolites with a VIP greater than 1 (as identified by OPLS-DA modelling of polar metabolites and FAME’s) and their corresponding coefficients can be seen in Table 1. Thirty-six polar metabolites were found to have a VIP greater than 1, with Extraction 2 samples having higher coefficients in the majority of cases, indicating comparatively higher levels of those metabolites (Table 1). Samples for Extraction 2 had positive coefficients for about half of these metabolites and Extraction 3 samples had negative coefficients in the majority of cases. Four fatty acids were found to have a VIP greater than 1, with Extraction 1 samples having positive coefficients for all 4 of the FAME’s; Extraction 2 samples have two positive and two negative coefficients and Extraction 3 samples having three negative coefficients (Table 1). Shared and unique structure (SUS) plots were also generated to compare similarities and differences in metabolites extracted by protocols 1 and 2 using samples from Extraction 3 as a common profile (Figure 3). The SUS plots for both the polar and FAME metabolites show that all metabolites identified to have a VIP > 1 score vary in the same direction and that neither protocol 1 or 2 extracts any unique metabolites compared to the other. This indicates that, other than differences in the levels of the metabolites extracted by the protocols, there is no difference in the breadth of polar and fatty acid metabolites recovered between the two extraction protocols.

**Table 1:** Metabolites identified to have a VIP score greater than 1 through OPLS-DA modelling of aqueous metabolite and FAME extraction data and the corresponding coefficient values for each extraction protocol. Values with a positive coefficient indicate higher comparative levels, while values with a negative coefficient indicate lower comparative levels.

Metabolite	VIP	Coefficient Extraction 1	Coefficient Extraction 2	Coefficient Extraction 3
Threonine	1.832	0.013	0.046	−0.059
Glycerol	1.703	−0.012	−0.042	0.054
Phenylalanine	1.678	0.030	0.034	−0.064
Alanine-3cyano	1.666	0.037	0.009	−0.046
Methionine	1.649	0.014	0.041	−0.055
Proline	1.630	−0.001	0.029	−0.028
Sorbitol	1.603	0.002	0.023	−0.024
Phosphoric Acid	1.575	−0.008	0.052	−0.043
Homoserine	1.531	−0.001	0.049	−0.048
Pyroglutamic Acid	1.496	0.002	0.014	−0.016
Alanine	1.476	−0.023	0.025	−0.002
Ornithine	1.461	−0.011	−0.046	0.057
Serine-O acetyl	1.382	−0.029	−0.035	0.064
Fumaric Acid	1.372	0.002	0.004	−0.006
Trehalose-alpha,alpha’-D	1.340	0.013	0.002	−0.015
Alanine-beta	1.327	0.007	0.006	−0.013
Succinic Acid	1.310	−0.006	−0.011	0.017
Malic acid, 2-isopropyl	1.299	0.016	0.020	−0.035
Decan-1-ol, n-	1.296	0.003	−0.004	0.002
Glycine	1.271	−0.027	−0.014	0.041
Valine	1.267	−0.010	0.033	−0.023
Aspartic Acid	1.261	0.016	0.012	−0.028
Arginine [-NH3]	1.259	0.030	0.003	−0.033
Glutamic Acid	1.213	0.009	0.007	−0.016
Hexadecane, n-	1.210	−0.016	0.036	−0.019
Malic Acid	1.190	0.005	0.002	−0.006
Uracil	1.189	−0.039	−0.017	0.056
Isoleucine	1.162	−0.009	0.019	−0.011
Glutamine, DL-	1.156	0.029	−0.023	−0.007
Octylamine	1.131	−0.005	0.005	−0.001
Tyramine	1.119	0.014	0.012	−0.026
Butanoic Acid	1.063	−0.010	0.019	−0.008
Serine	1.062	−0.016	0.036	−0.020
Pentadecane, n-	1.029	−0.015	0.022	−0.008
Citric Acid	1.024	0.018	0.005	−0.023
Heptadecan-1-ol	1.010	−0.011	0.011	−0.001
Palmitic Acid	1.950	0.581	−0.372	−0.182
Palmitoleic Acid	1.273	0.173	0.082	−0.221
Oleic Acid	1.239	0.123	0.066	−0.164
Stearic Acid	1.139	0.058	−0.197	0.121

### 2.3. Comparison of FAME and Aqueous Metabolite Profiles Obtained

PCA modelling of the FAME data from the organic layer(s) of the extraction protocols produced a relatively weak model based on the statistics *R*^2^ and *Q*^2^. This can perhaps be attributed to the fact that *S. cerevisae* does not have an overly complex fatty acid profile as it usually produces only even numbered chain length fatty acids and only three unsaturated fatty acids all of which are monounsaturated [28]. Despite this, some clustering was observed in this study—although the clustering is not as tight as that seen with the polar metabolites extracted (Figure 2). This is to be expected, as the fatty acid profile of yeast is not nearly as complex as that of its polar metabolites and small differences in levels between samples may be magnified leading to reduced variance. Somewhat surprisingly, the primary separation of the samples is based on extraction method used, as opposed to growth temperature—as was the case with the polar metabolite profiles. One could expect to see changes in the fatty acid composition as a result of the increase in growth temperature, although the temperature increase may not have been dramatic enough to cause the anticipated changes. Four fatty acids were identified as significantly altered (VIP ≥ 1), and examination of the corresponding coefficient values for each protocol suggests that samples from Extraction 1 have comparatively higher levels, while samples from Extraction 3 have comparatively lower levels, with samples from Extraction 2 falling in the middle.

With relatively few significantly altered fatty acids, we conclude that the aqueous component of the samples is more sensitive to the extraction system composition, although this may be a result of the diverse polar compounds analyzed and relatively narrow class of non-polar compounds since our analytical evaluation was not comprehensive. For example, the organic component of the extract may not be suitable for analysis of all lipid species due to the wide variety of headgroup chemistries and, likewise, highly polar compounds such as phosphorylated nucleotides may be measured sub-optimally from the aqueous phase. Furthermore, our analysis was limited to GC-MS analysis under specific derivatization conditions, providing some analytical constraints. In spite of these limitations, we estimate that this evaluation provides some guidance for a first-approach towards analysis of sample with mixed physiochemical analytes of interest.

### 2.4. Summary

Considering the two best procedures (Extractions 1 and 2)—as identified through multivariate projection methods—neither protocol extracts any unique metabolites and essentially all metabolites vary in the same direction for both protocols (Figure 3). This suggests that both protocols could serve as viable options for extraction of both polar and non-polar metabolites, though one would tend to prefer Extraction protocol 2, as the polar metabolite profile is more complex than that of the fatty acid profile and Extraction protocol 2 seems to be able to extract comparatively higher levels of these metabolites in a more reproducible manner. Furthermore, we were able to show that the use of multivariate projection methods is a viable method to compare and evaluate established extraction protocols for reproducibility and relative amount/breadth of metabolites recovered.

## 3. Experimental Section

### 3.1. Yeast Growth and Harvesting

Yeast *S. cerevisiae* strain BY4741 (MATa; his3∆1, leu2∆0, met15∆0 and ura3∆0) was grown in 1 L liquid cultures composed of 0.67% (*w*/*v*) YNB without ammonium sulphate (MP Biomedical, Solon OH, USA), 2% (*w*/*v*) dextrose, 0.002% (*w*/*v*) histidine, 0.003% (*w*/*v*) leucine, 0.002% (*w*/*v*) methionine and 0.002% (*w*/*v*) uracil in an incubated shaker at 30 °C or 37 °C with a rotation speed of 150 rpm to an OD_600_ of approximately 0.2/mL. Each sample consisted of approximately 10 OD of pelleted cells, which were washed twice with water to remove all growth media, flash frozen in liquid nitrogen to prevent further growth and/or metabolite turnover and stored at −80 °C until extraction by one of the three chloroform/methanol based extraction protocols.

### 3.2. Metabolite Extraction

#### 3.2.1. Extraction Protocol 1

Cells were extracted using a modified version of an extraction previously described by Zaremberg *et al.* [26]. Briefly, yeast cell pellets were re-suspended in 1 mL of CHCl_3_:MeOH (1:1) and transferred to 2 mL bead beater vials 1/8 filled with 0.5 mm acid washed beads. Bead beating was sustained for 60 seconds at 4 °C using the homogenize setting to break the cell walls, followed by transfer of the lysate to a 15 mL falcon tube. Beads were rinsed with 1 mL CHCl_3_/MeOH (2/1) which was then combined with the previously obtained cell lysate. Next 0.5 mL CHCl_3_/MeOH (2/1), 0.5 mL CHCl_3_ and 1.5 mL H_2_O were added, the contents vortexed and centrifuged at 2500 rpm for 10 minutes at 4 °C. The aqueous layer was then collected, the protein layer aspirated off and the tube spun again at 2500 rpm for 5 min at 4 °C. Remaining protein was aspirated and the organic layer was collected and retained. Organic and aqueous fractions were stored at −80 °C after being dried overnight in a speed vacuum or fume hood respectively.

#### 3.2.2. Extraction Protocol 2

Cells were extracted using a modified version of an extraction previously described by McCombie *et al.* [27]. Briefly yeast cell pellets were re-suspended with 300 μL CHCl_3_/MeOH (1/2) and transferred to a 2 mL bead beater vial 1/8 filled with 0.5 mm acid washed beads. Bead beating for 60 seconds at 4 °C using the homogenize setting to break the cell walls was followed by transfer of the lysate to a microcentrifuge tube and the beads were rinsed with 300 μL CHCl_3_/MeOH (1/2) which was combined with the previously obtained cell lysate. Next, 200 μL each of CHCl_3_ and H_2_O were added and the contents vortexed, followed by centrifugation at 14,000 rpm for 7 min at 4 °C. The aqueous layer was isolated and transferred to a new microcentrifuge tube, the protein layer aspirated off and the organic phase collected and saved. The aqueous layer was then centrifuged 14,000 rpm for 7 min at 4 °C and saved. Organic and aqueous fractions were dried down and stored as described above.

#### 3.2.3. Extraction Protocol 3

Cells were extracted using a modified version of an extraction previously described by Ejsing *et al.* [28]. Briefly yeast cell pellets were re-suspended with 300 μL 150 mM NH_4_HCO_3_ and transferred to a 2 mL bead beater vial 1/8 filled with 0.5 mm acid washed beads. Bead beating for 60 seconds at 4 °C using the homogenize setting to break the cell walls was followed by transfer of the lysate to an microcentrifuge tube and the beads rinsed with 300 μL 150 mM NH_4_HCO_3_. This was combined with the previously obtained cell lysate. Next 990 μL CHCl_3_/MeOH (17/1) was added to the microcentrifuge tube and subject to passive extraction (extraction on a benchtop at room temperature without any centrifugation) for 2 h at 20 °C. The upper phase was subsequently isolated and transferred to another microcentrifuge tube while the lower organic phase was collected and saved. This followed by passive extraction of the isolated upper phase for 2 h at 20 °C with 990 μL CHCl_3_/MeOH (2/1). The aqueous and organic layers were isolated and placed in separate microcentrifuge tubes, resulting in a total of two different organic fractions and one aqueous fraction. Organic and aqueous fractions were dried down and stored as described above.

### 3.3. Derivatization and Sample Preparation

Aqueous samples were prepared for GC-MS analysis by derivatization with methoxyamine and MSTFA (*N*-methyl-*N*-(trimethylsilyl) trifluoroacetamide) as previously described [2]. Briefly 50 μL of 20 mg/mL solution of methoxylamine-hydrochloride in pyridine was added to each dry sample, with shaking at 37 °C for 2.5 h. 50 μL of MSTFA was then added and followed by 45 min of additional shaking at 37 °C.

Organic samples were prepared for GC-MS fatty acid methyl ester (FAME) analysis by derivitzation with BF_3_/methanol as previously described [27]. Briefly the dried down organic fractions were dissolved in 750 μL of 1:1 (CHCl_3_:MeOH ) under sonication for 15 minutes, followed by addition of 125 μL of BF_3_/methanol and incubation of the samples in glass vials at 80 °C for 90 min. After cooling, 300 μL of H_2_O and 600 μL of hexane were added to each sample and vortexed. The organic layer was then evaporated to dryness overnight in a fume hood.

These derivitization methods were used as they are most commonly found in literature and are well established. After derivitization samples were reconstituted in hexane.

### 3.4. GC-MS Data Acquisition

GC-MS acquisition was carried out using a Waters Micromass GCT Premier GC-TOF-MS (Waters, Missisauga Ontario, Canada) coupled to a 7683 B Series Injector and Autosampler (Agilent Technologies, Missisauga Ontario, Canada) with a 1 μL injection volume. For aqueous metabolite analysis an electron ionization (EI) source was used with a DB-5MS 30 m × 0.25 mm column with a 0.25 μm filament size (Agilent Technologies, Missisauga Ontario, Canada). For FAME analysis an EI source was used with a DB-23 60 m × 0.25 mm column with a 0.15 μm filament size (Agilent Technologies, Missisauga Ontario, Canada). The settings on the GC-MS were as follows: 275 °C and 240 °C injector temperature for the aqueous column and FAME column respectively, and a flow rate of helium (carrier gas) of 1.2 mL/min. Samples were run in a randomized order.

### 3.5. Data Processing and Interpretation

Raw GC-MS data was imported to MetaboliteDetector [29] for peak detection. The data were first normalized using Excel 2010 (Microsoft, Redmond, WA, USA) to the internal standard, D-25 Tridecanoic Acid, in the case of targeted FAME profiling, followed by integral normalization. For the untargeted polar metabolite profiling integral normalization was carried out. In Metabolite Detector for compound detection the peak threshold, minimal peak height and bins/scan parameters were all set at 2.00 and the deconvolution width was set at 1.90. For metabolite detection the values were set as 15.0, 0.30 and 0.70 for Maximum retention index (RI) difference, Pure/Impure Composition and Cutoff score respectively. Lastly, using the batch quantification tool integrated GCMS analysis was done. For this step the parameters for compound matching were set as 5.0, 0.30 and 0.85 for ΔRI, Pure/Impure and required similarity score (Req. Score) respectively, for identification 15.0 and 0.30 for ΔRI and Pure/Impure and for compound filter the signal to noise (S/N) parameter was set as 0.30. After normalization and compound detection, the data were exported to SIMCA-P13 (MKS Umetrics AB, Umea, Sweden), a multivariate statistical analytical software, where univariate scaling and mean centering was applied before the model construction and validation step. Model construction was done using the autofit routine of SIMCA-P, to avoid overfitting of the data, and the OPLS-DA models were validated with CV-ANOVA *p*-values.

## 4. Conclusions

Choosing the correct extraction protocol for a given organism or biological system is of fundamental importance when designing metabolomic studies as the metabolite extraction step has a direct effect on all subsequent steps of data collection and analysis. Using multivariate projection methods, we were able to compare three established chloroform/methanol/water partitioning metabolite extraction methods for the ability to reproducibly extract both polar and non-polar yeast metabolites. Using this approach a highly reproducible method was identified that was able to extract comparatively higher amounts of polar metabolites—all three protocols were able to obtain comparable metabolite breadths. We were able to confirm the effectiveness of chemoinformatics and multivariate projection methods to efficiently give a comparison of different extraction protocols for a given organism. This approach should prove an efficient way to compare other established extraction protocols for other systems against each other, as well as providing a quicker and more cost effective way of comparing new extraction protocols to previously established ones.
